# Supplementary material for: A Likelihood Approach for Real-Time Calibration of Stochastic Compartmental Epidemic Models
Source: PLoS Comput Biol. 2017 Jan 17;13(1):e1005257. doi: 10.1371/journal.pcbi.1005257 (PMC5240920; doi:10.1371/journal.pcbi.1005257)
Supplement: S1 File — (TAR.GZ) [file pcbi.1005257.s014.tar.gz › HSPH_Online-SI-Revision/MSS10/n10-extreme/n10-extreme_NEW2_table-8.pdf]

1	newly 3 weeks spec	116.	180.82	{25., 413.}	98.6724	0.411553	newly 3 weeks cumul	174.	285.78	{44., 632.}	99.4828	0.343659	newly 5 weeks spec	547.	684.99	{100., 1380.}	73.6508	0.42507	newly 5 weeks cumul	988.	1361.71	{202., 2868.}	82.7905	0.37599	newly 66 weeks cumul	9069.	8062.53	{5715., 9247.}	12.1119	0.148976
2	newly 3 weeks spec	211.	149.61	{15., 290.}	52.2322	0.504722	newly 3 weeks cumul	450.	370.92	{79., 665.}	45.4578	0.335722	newly 5 weeks spec	408.	206.77	{0., 452.}	58.826	0.808991	newly 5 weeks cumul	1168.	763.05	{109., 1509.}	50.3981	0.446118	newly 62 weeks cumul	7262.	2428.62	{109., 5341.}	67.9229	0.790336
3	newly 3 weeks spec	63.	30.61	{0., 35.}	111.54	1.44055	newly 3 weeks cumul	84.	43.89	{1., 56.}	107.417	1.10652	newly 5 weeks spec	540.	169.35	{0., 316.}	102.539	2.00341	newly 5 weeks cumul	830.	305.07	{2., 436.}	105.912	1.60717	newly 68 weeks cumul	9108.	5210.08	{2., 9248.}	43.4297	1.15289
4	newly 3 weeks spec	319.	356.1	{209., 495.}	31.6552	0.144243	newly 3 weeks cumul	641.	686.41	{452., 915.}	27.0936	0.126343	newly 5 weeks spec	626.	737.28	{511., 979.}	30.3834	0.130239	newly 5 weeks cumul	1754.	1972.94	{1305., 2649.}	28.447	0.126751	newly 62 weeks cumul	8332.	8121.44	{7350., 9190.}	10.186	0.0526324
5	newly 3 weeks spec	240.	211.62	{59., 346.}	47.6333	0.294563	newly 3 weeks cumul	440.	382.81	{108., 640.}	43.6932	0.249801	newly 5 weeks spec	689.	538.17	{182., 901.}	36.8345	0.318182	newly 5 weeks cumul	1573.	1282.54	{449., 2088.}	39.6402	0.263984	newly 65 weeks cumul	9428.	6832.4	{3695., 9286.}	27.5348	0.239306
6	newly 3 weeks spec	128.	293.33	{56., 547.}	150.805	0.404517	newly 3 weeks cumul	239.	480.45	{102., 885.}	121.418	0.344744	newly 5 weeks spec	489.	880.	{219., 1588.}	101.599	0.364856	newly 5 weeks cumul	1036.	1931.04	{504., 3373.}	108.066	0.344326	newly 66 weeks cumul	9287.	7030.92	{3833., 9183.}	24.3622	0.165458
7	newly 3 weeks spec	131.	295.54	{3., 986.}	200.687	0.728458	newly 3 weeks cumul	207.	475.42	{24., 1297.}	192.251	0.501893	newly 5 weeks spec	469.	445.75	{0., 1228.}	87.162	0.952872	newly 5 weeks cumul	924.	1341.87	{24., 4517.}	125.811	0.623362	newly 67 weeks cumul	8335.	4004.43	{24., 9004.}	54.7125	0.763021
8	newly 3 weeks spec	187.	329.86	{133., 625.}	92.6845	0.315863	newly 3 weeks cumul	315.	584.89	{234., 1063.}	97.673	0.283668	newly 5 weeks spec	589.	802.57	{347., 1291.}	56.0526	0.28877	newly 5 weeks cumul	1255.	1945.57	{924., 3337.}	70.4056	0.259516	newly 64 weeks cumul	9223.	7182.72	{3581., 9284.}	22.3585	0.205009
9	newly 3 weeks spec	245.	358.83	{211., 540.}	51.7673	0.16388	newly 3 weeks cumul	451.	619.35	{386., 915.}	43.612	0.144385	newly 5 weeks spec	556.	967.33	{637., 1265.}	74.4514	0.230252	newly 5 weeks cumul	1389.	2228.17	{1366., 3105.}	61.6004	0.193844	newly 64 weeks cumul	8874.	8519.58	{7588., 8986.}	5.19247	0.0244579
10	newly 3 weeks spec	219.	223.69	{11., 475.}	67.3288	0.549537	newly 3 weeks cumul	414.	439.56	{55., 845.}	62.6522	0.382984	newly 5 weeks spec	476.	419.74	{2., 937.}	58.7227	0.64449	newly 5 weeks cumul	1223.	1187.16	{89., 2469.}	59.8299	0.439038	newly 64 weeks cumul	8927.	3924.44	{93., 7317.}	56.1893	0.617648
11	newly 3 weeks spec	260.	126.66	{61., 154.}	59.6308	0.393487	newly 3 weeks cumul	477.	262.58	{147., 311.}	52.5493	0.321467	newly 5 weeks spec	539.	242.49	{112., 334.}	59.8534	0.421036	newly 5 weeks cumul	1360.	688.44	{340., 857.}	56.7485	0.369091	newly 59 weeks cumul	8770.	7103.72	{5762., 8372.}	18.9998	0.099512
12	newly 3 weeks spec	147.	109.78	{41., 186.}	37.9048	0.237971	newly 3 weeks cumul	271.	202.82	{90., 330.}	34.6125	0.206029	newly 5 weeks spec	376.	325.27	{96., 568.}	40.9282	0.267277	newly 5 weeks cumul	836.	720.84	{270., 1180.}	37.5837	0.216362	newly 64 weeks cumul	8374.	7863.68	{5027., 9268.}	12.1674	0.07775
13	newly 3 weeks spec	356.	224.25	{124., 305.}	41.2388	0.244103	newly 3 weeks cumul	666.	456.13	{285., 578.}	36.1577	0.203062	newly 5 weeks spec	681.	462.76	{280., 625.}	33.3392	0.193238	newly 5 weeks cumul	1797.	1256.96	{752., 1690.}	32.9338	0.186434	newly 60 weeks cumul	8214.	7324.46	{6543., 8494.}	12.5405	0.0623066
14	newly 3 weeks spec	267.	469.31	{161., 731.}	93.4719	0.290526	newly 3 weeks cumul	466.	751.91	{238., 1202.}	79.7532	0.261703	newly 5 weeks spec	683.	1224.1	{693., 1687.}	88.8814	0.288894	newly 5 weeks cumul	1583.	2846.39	{1336., 4156.}	91.0783	0.284734	newly 65 weeks cumul	8671.	8207.54	{6799., 9323.}	10.3912	0.0566688
15	newly 3 weeks spec	284.	165.85	{114., 215.}	41.6092	0.24921	newly 3 weeks cumul	552.	339.74	{228., 442.}	38.4529	0.223362	newly 5 weeks spec	600.	382.83	{289., 483.}	36.195	0.207095	newly 5 weeks cumul	1572.	981.11	{724., 1248.}	37.5884	0.216737	newly 62 weeks cumul	7388.	8517.46	{7933., 8866.}	15.4751	0.0620728
16	newly 3 weeks spec	140.	222.87	{61., 508.}	92.7214	0.27757	newly 3 weeks cumul	241.	341.85	{105., 736.}	76.6515	0.25285	newly 5 weeks spec	517.	793.2	{245., 1737.}	82.4062	0.272509	newly 5 weeks cumul	1029.	1606.27	{434., 3430.}	86.0845	0.271748	newly 65 weeks cumul	8875.	8924.79	{8310., 9375.}	4.15944	0.0181901
17	newly 3 weeks spec	117.	182.06	{7., 341.}	145.094	0.613504	newly 3 weeks cumul	203.	305.27	{25., 507.}	136.704	0.469441	newly 5 weeks spec	499.	341.95	{12., 887.}	76.7595	0.778185	newly 5 weeks cumul	983.	892.62	{55., 2117.}	91.9288	0.592184	newly 67 weeks cumul	8786.	5922.38	{501., 9254.}	34.6688	0.406846
18	newly 3 weeks spec	167.	357.1	{154., 572.}	121.772	0.326511	newly 3 weeks cumul	309.	588.44	{280., 909.}	98.0971	0.280089	newly 5 weeks spec	657.	1038.25	{477., 1503.}	69.137	0.231103	newly 5 weeks cumul	1340.	2312.92	{1111., 3407.}	81.2821	0.24962	newly 65 weeks cumul	9346.	7926.74	{6183., 9151.}	15.2012	0.0802545
19	newly 3 weeks spec	61.	34.29	{1., 74.}	73.1639	0.738912	newly 3 weeks cumul	87.	56.88	{5., 119.}	69.4483	0.544889	newly 5 weeks spec	390.	159.38	{1., 329.}	81.159	1.06621	newly 5 weeks cumul	643.	295.38	{10., 594.}	78.5163	0.842518	newly 67 weeks cumul	8626.	7101.11	{235., 9283.}	21.1668	0.385139
20	newly 3 weeks spec	181.	120.97	{43., 194.}	38.547	0.264424	newly 3 weeks cumul	336.	219.79	{96., 341.}	37.9435	0.249707	newly 5 weeks spec	555.	365.57	{105., 640.}	41.418	0.295129	newly 5 weeks cumul	1224.	802.99	{283., 1359.}	40.0025	0.27633	newly 64 weeks cumul	9145.	8048.76	{5752., 9297.}	12.5382	0.0633172
21	newly 3 weeks spec	233.	283.51	{179., 375.}	32.0215	0.121755	newly 3 weeks cumul	468.	540.64	{336., 700.}	26.5128	0.104339	newly 5 weeks spec	546.	638.54	{381., 815.}	29.0733	0.116547	newly 5 weeks cumul	1440.	1631.52	{1007., 2111.}	26.0236	0.106135	newly 61 weeks cumul	8562.	8715.55	{8077., 9263.}	4.76302	0.020461
22	newly 3 weeks spec	42.	64.46	{0., 56.}	190.714	1.09186	newly 3 weeks cumul	65.	92.29	{2., 88.}	171.492	0.80029	newly 5 weeks spec	339.	152.66	{0., 360.}	103.386	1.67762	newly 5 weeks cumul	532.	349.45	{2., 605.}	118.731	1.24354	newly 68 weeks cumul	9331.	5075.09	{2., 9286.}	45.6955	1.07912
23	newly 3 weeks spec	289.	257.05	{40., 504.}	52.3287	0.394924	newly 3 weeks cumul	567.	544.23	{107., 1021.}	51.6526	0.311204	newly 5 weeks spec	502.	374.09	{54., 619.}	43.6315	0.420353	newly 5 weeks cumul	1436.	1248.86	{243., 2202.}	46.8997	0.319566	newly 63 weeks cumul	6906.	3258.55	{976., 4170.}	53.0552	0.436705
24	newly 3 weeks spec	396.	651.49	{554., 735.}	64.5177	0.213111	newly 3 weeks cumul	794.	1263.77	{1068., 1455.}	59.165	0.198832	newly 5 weeks spec	721.	1098.66	{1019., 1182.}	52.38	0.182254	newly 5 weeks cumul	2068.	3282.2	{2953., 3587.}	58.7137	0.199115	newly 61 weeks cumul	8926.	8695.96	{8165., 8934.}	2.96079	0.013334
25	newly 3 weeks spec	77.	10.33	{1., 21.}	87.987	1.16085	newly 3 weeks cumul	116.	22.53	{4., 44.}	82.5259	0.883674	newly 5 weeks spec	441.	25.06	{1., 50.}	94.3175	1.67801	newly 5 weeks cumul	781.	64.26	{8., 131.}	91.8643	1.33692	newly 64 weeks cumul	9000.	5616.47	{15., 8202.}	37.5948	0.579859
26	newly 3 weeks spec	130.	1452.53	{34., 3618.}	1045.84	1.07974	newly 3 weeks cumul	214.	2080.09	{86., 5131.}	897.005	0.900494	newly 5 weeks spec	577.	1395.04	{43., 2278.}	175.629	0.689553	newly 5 weeks cumul	1076.	5231.95	{226., 8762.}	415.334	0.809882	newly 67 weeks cumul	7335.	7643.94	{1415., 9386.}	30.1832	0.31402
27	newly 3 weeks spec	133.	133.37	{41., 217.}	49.4511	0.270811	newly 3 weeks cumul	225.	217.86	{74., 354.}	44.3467	0.223172	newly 5 weeks spec	482.	513.36	{133., 884.}	52.5851	0.286322	newly 5 weeks cumul	995.	1011.23	{322., 1741.}	49.392	0.255073	newly 65 weeks cumul	9083.	8451.9	{7668., 9316.}	7.95486	0.0612178
28	newly 3 weeks spec	69.	573.56	{5., 1616.}	779.304	0.865693	newly 3 weeks cumul	105.	857.92	{14., 3405.}	760.762	0.696064	newly 5 weeks spec	530.	860.57	{4., 2143.}	128.617	0.809551	newly 5 weeks cumul	861.	2472.17	{28., 6942.}	245.627	0.721557	newly 68 weeks cumul	9312.	6678.76	{28., 9282.}	28.3791	0.445408
29	newly 3 weeks spec	156.	68.	{22., 102.}	68.4872	0.504115	newly 3 weeks cumul	228.	123.79	{49., 190.}	56.9079	0.374629	newly 5 weeks spec	612.	187.12	{53., 325.}	75.0033	0.63933	newly 5 weeks cumul	1151.	433.75	{139., 699.}	71.0643	0.551372	newly 63 weeks cumul	9014.	8549.31	{7816., 9096.}	5.59696	0.0256121
30	newly 3 weeks spec	209.	448.11	{241., 817.}	116.962	0.298446	newly 3 weeks cumul	375.	780.5	{446., 1353.}	110.363	0.292253	newly 5 weeks spec	521.	995.69	{630., 1551.}	93.9981	0.273655	newly 5 weeks cumul	1271.	2520.17	{1530., 4032.}	101.027	0.28109	newly 63 weeks cumul	8143.	8312.54	{6907., 9296.}	11.7944	0.054681
31	newly 3 weeks spec	173.	819.57	{249., 1348.}	377.012	0.641786	newly 3 weeks cumul	303.	1226.95	{379., 1985.}	308.327	0.558252	newly 5 weeks spec	607.	1672.52	{1096., 1994.}	181.542	0.48225	newly 5 weeks cumul	1297.	4340.42	{2111., 5999.}	239.315	0.524158	newly 66 weeks cumul	9186.	8441.95	{6074., 9376.}	9.40094	0.0744979
32	newly 3 weeks spec	258.	192.16	{160., 230.}	25.5194	0.132175	newly 3 weeks cumul	565.	402.54	{345., 468.}	28.754	0.149952	newly 5 weeks spec	577.	384.88	{325., 443.}	33.2964	0.179245	newly 5 weeks cumul	1557.	1066.05	{933., 1211.}	31.5318	0.167126	newly 58 weeks cumul	8222.	8366.88	{8272., 8501.}	2.63658	0.0114156
33	newly 3 weeks spec	126.	69.91	{4., 134.}	86.5635	0.793182	newly 3 weeks cumul	184.	119.1	{10., 230.}	84.4674	0.62575	newly 5 weeks spec	733.	194.97	{4., 417.}	79.9986	1.06688	newly 5 weeks cumul	1177.	435.25	{22., 1082.}	78.8352	0.854405	newly 66 weeks cumul	9187.	7380.11	{3795., 9271.}	20.0563	0.218257
34	newly 3 weeks spec	135.	2394.94	{170., 3978.}	1687.24	1.19206	newly 3 weeks cumul	190.	4070.27	{316., 7399.}	2052.48	1.20655	newly 5 weeks spec	568.	867.98	{25., 1736.}	101.095	0.552443	newly 5 weeks cumul	1068.	6684.5	{1077., 9095.}	539.71	0.83602	newly 68 weeks cumul	8375.	7471.15	{3982., 9364.}	18.7913	0.179751
35	newly 3 weeks spec	297.	325.2	{208., 446.}	25.8316	0.109087	newly 3 weeks cumul	580.	605.39	{402., 813.}	21.5362	0.0932441	newly 5 weeks spec	563.	742.35	{506., 957.}	37.8845	0.137971	newly 5 weeks cumul	1559.	1865.35	{1221., 2401.}	28.8768	0.111961	newly 63 weeks cumul	8157.	8222.98	{6776., 9223.}	10.7596	0.0493923
36	newly 3 weeks spec	233.	153.88	{90., 208.}	34.4206	0.206322	newly 3 weeks cumul	424.	307.32	{201., 397.}	28.0142	0.15924	newly 5 weeks spec	562.	345.32	{215., 474.}	38.5658	0.239209	newly 5 weeks cumul	1395.	892.51	{573., 1188.}	36.0867	0.215879	newly 61 weeks cumul	9215.	7434.94	{6662., 8731.}	19.317	0.0978958
37	newly 3 weeks spec	197.	85.45	{17., 101.}	82.7259	0.634935	newly 3 weeks cumul	298.	140.5	{34., 176.}	76.0336	0.537517	newly 5 weeks spec	605.	231.97	{45., 351.}	72.6264	0.650832	newly 5 weeks cumul	1277.	524.34	{107., 713.}	75.823	0.610447	newly 64 weeks cumul	9224.	8596.32	{7847., 9348.}	7.48157	0.0558548
38	newly 3 weeks spec	40.	26.73	{0., 65.}	90.025	0.888739	newly 3 weeks cumul	62.	44.58	{3., 113.}	85.5806	0.662525	newly 5 weeks spec	491.	128.73	{0., 400.}	84.2179	1.46338	newly 5 weeks cumul	699.	234.78	{5., 668.}	83.2818	1.11311	newly 67 weeks cumul	8849.	6317.7	{12., 9066.}	29.9801	0.598996
39	newly 3 weeks spec	103.	15.88	{0., 38.}	88.8155	1.39602	newly 3 weeks cumul	146.	26.51	{2., 60.}	86.2534	1.10514	newly 5 weeks spec	645.	62.55	{0., 119.}	93.6822	1.94374	newly 5 weeks cumul	1060.	120.94	{2., 260.}	92.2698	1.54222	newly 67 weeks cumul	9368.	6037.95	{3., 9196.}	35.5879	0.809038
40	newly 3 weeks spec	276.	586.76	{352., 809.}	113.87	0.311523	newly 3 weeks cumul	519.	1029.17	{641., 1401.}	100.033	0.284779	newly 5 weeks spec	547.	1198.89	{860., 1477.}	121.04	0.335351	newly 5 weeks cumul	1464.	3172.74	{2137., 4039.}	117.5	0.324197	newly 64 weeks cumul	8627.	8091.73	{5700., 9101.}	10.0103	0.0533158
41	newly 3 weeks spec	249.	163.15	{70., 267.}	50.7269	0.309421	newly 3 weeks cumul	416.	309.37	{146., 504.}	44.2957	0.246831	newly 5 weeks spec	680.	397.4	{159., 689.}	49.1941	0.328457	newly 5 weeks cumul	1527.	971.94	{446., 1619.}	48.1493	0.301284	newly 63 weeks cumul	9091.	7746.8	{5331., 9032.}	14.9841	0.0854568
42	newly 3 weeks spec	386.	295.48	{191., 431.}	31.3731	0.170394	newly 3 weeks cumul	794.	618.66	{418., 877.}	29.	0.153688	newly 5 weeks spec	673.	527.73	{349., 758.}	28.4413	0.15191	newly 5 weeks cumul	2037.	1555.08	{1037., 2208.}	29.2086	0.158171	newly 58 weeks cumul	8720.	7535.51	{6862., 8908.}	14.687	0.0715836
43	newly 3 weeks spec	32.	27.61	{0., 28.}	139.344	1.18503	newly 3 weeks cumul	43.	39.08	{1., 42.}	129.442	0.839149	newly 5 weeks spec	315.	107.9	{0., 93.}	108.114	1.88354	newly 5 weeks cumul	461.	213.99	{2., 174.}	114.445	1.40616	newly 68 weeks cumul	9060.	5243.7	{2., 9148.}	42.6898	1.04115
44	newly 3 weeks spec	255.	269.77	{171., 323.}	20.2784	0.0902977	newly 3 weeks cumul	527.	537.69	{363., 650.}	16.8102	0.076332	newly 5 weeks spec	597.	556.09	{354., 686.}	15.6566	0.0812477	newly 5 weeks cumul	1543.	1499.53	{981., 1835.}	16.3973	0.0799072	newly 59 weeks cumul	8693.	8597.04	{7150., 9037.}	6.16036	0.0284863
45	newly 3 weeks spec	293.	458.47	{259., 779.}	63.2389	0.199664	newly 3 weeks cumul	562.	826.47	{496., 1280.}	54.3612	0.176764	newly 5 weeks spec	606.	937.26	{673., 1222.}	57.6601	0.201781	newly 5 weeks cumul	1576.	2488.27	{1646., 3623.}	61.3065	0.200903	newly 63 weeks cumul	8765.	8199.36	{5961., 9265.}	10.1684	0.0519886
46	newly 3 weeks spec	273.	290.22	{204., 383.}	20.674	0.0884778	newly 3 weeks cumul	518.	563.09	{414., 721.}	19.3958	0.0808705	newly 5 weeks spec	572.	654.47	{458., 851.}	22.8969	0.0905833	newly 5 weeks cumul	1495.	1676.35	{1208., 2166.}	21.2502	0.0854542	newly 61 weeks cumul	8858.	8454.3	{8016., 8920.}	5.17882	0.0249777
47	newly 3 weeks spec	140.	95.03	{43., 148.}	39.2071	0.27167	newly 3 weeks cumul	227.	177.38	{87., 259.}	33.2775	0.197308	newly 5 weeks spec	505.	282.09	{110., 478.}	46.2158	0.347867	newly 5 weeks cumul	1027.	627.63	{271., 1006.}	42.4625	0.290375	newly 63 weeks cumul	9302.	8306.84	{7326., 9273.}	10.7463	0.0767777
48	newly 3 weeks spec	115.	207.23	{30., 492.}	119.522	0.42127	newly 3 weeks cumul	204.	329.66	{76., 737.}	98.9706	0.318006	newly 5 weeks spec	483.	705.52	{69., 1534.}	90.6791	0.525303	newly 5 weeks cumul	959.	1467.61	{189., 3353.}	96.0719	0.382189	newly 66 weeks cumul	9126.	7595.3	{2577., 9364.}	18.1407	0.255627
49	newly 3 weeks spec	85.	28.09	{0., 49.}	91.6118	1.20947	newly 3 weeks cumul	133.	46.35	{3., 82.}	87.7368	0.942273	newly 5 weeks spec	380.	98.15	{0., 196.}	92.8658	1.53769	newly 5 weeks cumul	694.	203.38	{5., 372.}	92.4841	1.20479	newly 67 weeks cumul	8859.	5804.2	{6., 9072.}	35.5997	0.741234
50	newly 3 weeks spec	88.	55.86	{10., 123.}	58.6136	0.465073	newly 3 weeks cumul	156.	104.77	{26., 215.}	53.8141	0.380603	newly 5 weeks spec	376.	175.51	{12., 402.}	62.6463	0.657874	newly 5 weeks cumul	733.	383.7	{54., 876.}	60.0409	0.525754	newly 65 weeks cumul	9078.	7208.71	{3571., 9135.}	21.1786	0.243787
